# Combined submandibular gland flap and sternocleidomastoid musculocutaneous flap for postoperative reconstruction in older aged patients with oral cavity and oropharyngeal cancers

**DOI:** 10.1186/1477-7819-12-259

**Published:** 2014-08-15

**Authors:** Xiangmin Zhang, Folin Liu, Xiaolin Lan, Keqing Luo, Shaojin Li

**Affiliations:** Department of Head and Neck Surgery, Ganzhou Tumor Hospital, Ganzhou, Jiangxi Province People’s Republic of China; First Affiliated Hospital of Gannan Medical University, Ganzhou, Jiangxi Province People’s Republic of China; Ganzhou Institute of Cancer Research, 19, HuaYuan Qian Road, Ganzhou, 341000 Jiangxi Province People’s Republic of China

**Keywords:** Submandibular gland flap, Sternocleidomastoid musculocutaneous flap, Oral cavity cancer, Oropharyngeal cancer, Reconstruction

## Abstract

**Background:**

The growth of aging populations in an increasing number of countries has led to a concomitant increase in the incidence of chronic diseases. Accordingly, the proportion of older aged patients with oral cavity and oropharyngeal cancers and comorbidities has also increased. Thus, improvements must be made in the tolerance and safety of surgical procedures for these patients with complex medical conditions. In this study, we investigated combined submandibular gland flap and sternocleidomastoid musculocutaneous flap for postoperative reconstruction in older aged patients with oral cavity and oropharyngeal cancers in terms of surgical methods, safety, and clinical outcome.

**Methods:**

Between January 2011 and May 2012, 8 patients over the age of 65 years (7 men, 1 woman; aged 66 to 75 years (median, 69.6)) with oral cavity and oropharyngeal cancers underwent combined submandibular gland and sternocleidomastoid myocutaneous flaps for postoperative reconstruction at Ganzhou Tumor Hospital. All eight patients had comorbid cardiovascular, cerebrovascular, or chronic respiratory disease or diabetes. Clinical outcomes, complications, and tolerance to surgical treatment were observed.

**Results:**

Surgical treatment was successful in all eight patients. All submandibular gland flaps survived with well-mucosalized surfaces and with no complications. During the postoperative follow-up period of 12 to 28 months, no patient developed local recurrence or distant metastasis, and all had good recovery of function and local contour.

**Conclusions:**

This combined reconstruction technique enables appropriate restoration of oral function, facial aesthetics and improved quality of life. Further, this technique has several advantages: it is easier to perform, reduces operation time and surgical risk, causes less surgical injury, and has minor impact on contour. The technique provides a new and safe reconstruction option for older aged patients with oral cavity and oropharyngeal cancers.

## Background

A number of major organs are concentrated in the oral cavity and oropharynx. Surgical removal of oral cavity and oropharyngeal cancers can lead to facial injury or even disfigurement and decreased oral functions, including speech, mastication, and swallowing. Surgical reconstruction with skin flap, musculocutaneous flap, and/or osteo-myocutaneous flap enables oral cancer patients to achieve appropriate restoration of oral function and facial aesthetics and a greatly improved quality of life [1, 2]. With the gradual aging of society, the proportion of older aged patients with oral cavity and oropharyngeal cancers and comorbid cardiovascular disease, diabetes, chronic respiratory disease, or cerebrovascular disease has been markedly increasing. Older aged patients with complex medical conditions may have poor surgical tolerance and be unable to undergo lengthy and risky operative procedures. Conventional reconstructive surgeries with skin, musculocutaneous, and osteo-myocutaneous flaps tend to be time-consuming and cause relatively large operative trauma, thus potentially increasing surgical risk. Moreover, the postoperative recovery of older aged patients, especially those with comorbid systemic disease, may be delayed. Therefore, there is an increasing need for a new reconstruction method to improve the tolerance and safety of reconstructive surgery [3].

Oral cavity and oropharyngeal cancers most often metastasize to neck lymph nodes, and neck lymphatic metastases are most frequently observed in levels I, II, and III [4]. The submandibular glands are located in level Ib, where they are surrounded by rich lymphatic tissues. However, the involvement of submandibular glands in oral cavity and oropharyngeal cancers is quite rare, especially in early oral cancers [5, 6]. Additionally, the existence of intraglandular lymph nodes within submandibular glands continues to be debated [7, 8]. Studies have demonstrated that preservation of the submandibular gland during neck dissection is oncologically safe in patients with early oral cancers unless the primary tumor or metastatic regional lymphadenopathy is adherent to the gland [9, 10]. It has also been reported that the submandibular gland can survive and maintain its integrity and function following surgical transfer and reimplantation [11, 12]. These studies suggest that the submandibular gland can be used in the surgical reconstruction of oral defects. Therefore, it is of clinical significance to explore further the feasibility of the submandibular gland flap for postoperative reconstruction in patients with early oral cavity and oropharyngeal cancers.

Between January 2011 and May 2012, we performed reconstructive surgeries for oral defects using combined submandibular gland and sternocleidomastoid musculocutaneous flaps in eight older aged patients with oral cavity and oropharyngeal cancers at our facility. In this study, we conducted a retrospective analysis of these patients and report on the safety, practicality, and clinical effects of this new reconstruction method.

## Methods

### Ethics statement

The ethics committee of the Tumor Hospital of Ganzhou Review Board approved the study protocol (20101201), and the study was conducted in accordance with the principles of the Declaration of Helsinki regarding research involving human subjects. Each of the patients provided written informed consent to participate after the nature of the study had been explained to them.

### Inclusion and exclusion criteria

Inclusion criteria: (1) over 65 years old; (2) pathologically confirmed diagnosis of oral cavity cancer or oropharyngeal cancer; (3) no cervical lymph node metastasis or no level I lymphatic metastasis; (4) tumor staging: < T3; (5) comorbid cardiovascular disease, diabetes, chronic respiratory disease, or cerebrovascular disease; (6) Karnofsky score: ≥ 80; (7) expected survival: over 1 year; (8) voluntarily signed informed consent. Exclusion criteria: (1) bilateral cervical lymph node metastasis; (2) bilateral level I cervical lymph node metastasis; (3) diseased submandibular gland; (4) prior neck surgery or neck radiotherapy; (5) distant metastasis; (6) unable to tolerate surgical operation due to poor clinical condition.

### Clinical data

Between January 2011 and May 2012, combined submandibular gland and sternocleidomastoid myocutaneous flap postoperative reconstruction was performed in eight patients over 65 years of age (seven men, one woman; age, 66 to 75 years (median, 69.6)) with oral cavity and oropharyngeal cancers at Ganzhou Tumor Hospital. Comorbidities included three cases of hypertension, two cases of chronic bronchitis, two cases of diabetes mellitus, and one case of history of cerebral infarction. Detailed clinical data are presented in Table 1.

**Table 1:** **Clinical data**

Case number	Sex	Age (years)	Site of primary tumor	Tumor dimensions (cm)	Pathology	Extent of defect (cm)	Comorbidities	Procedure	Repair method
1	Male	68	Lateral border of tongue	4 × 3	Squamous	6 × 5	Primary hypertension	Tongue carcinoma resection plus total neck dissection	Tongue defects repaired with submandibular gland flap; floor of mouth repaired with sternocleidomastoid muscle flap
2	Female	66	Lateral border of tongue	3 × 3	Squamous	5 × 5	Diabetes mellitus	Tongue carcinoma resection plus total neck dissection	Tongue defects repaired with submandibular gland flap; floor of mouth repaired with sternocleidomastoid muscle flap
3	Male	75	Lateral border of tongue	3 × 2	Squamous	5 × 4	Chronic bronchitis	Tongue carcinoma resection plus total neck dissection	Tongue defects repaired with submandibular gland flap; floor of mouth repaired with sternocleidomastoid muscle flap
4	Male	71	Root of tongue	2 × 2	Squamous	4 × 4	Primary hypertension	Root-of-tongue carcinoma resection plus whole neck dissection	Tongue-root defects repaired with submandibular gland flap; floor of mouth repaired with sternocleidomastoid muscle flap
5	Male	67	Root of tongue	3 × 2	Squamous	5 × 4	Cerebral infarction	Root-of-tongue carcinoma resection plus whole neck dissection	Tongue-root defects repaired with submandibular gland flap; floor of mouth repaired with sternocleidomastoid muscle flap
6	Male	70	Floor of mouth	2 × 2	Squamous	4 × 4	Primary hypertension	Floor-of-mouth cancer resection plus whole neck dissection	Floor-of-mouth mucosal defects repaired with submandibular gland flap; floor of mouth repaired with sternocleidomastoid muscle flap
7	Male	78	Gingiva	3 × 3	Squamous	5 × 5	Chronic bronchitis	Mandible segmental resection plus total neck dissection	Mandibular defects repaired with submandibular flap; floor of mouth repaired with sternocleidomastoid muscle flap
8	Male	72	Gingiva	4 × 3	Squamous	6 × 5	Diabetes mellitus	Mandible segmental resection plus total neck dissection	Mandibular defects repaired with submandibular flap; floor of mouth repaired with sternocleidomastoid muscle flap

### Surgical procedure

The surgeries were performed under general anesthesia. Ipsilateral primary tumor resection and total neck level I to V lymph node dissection were performed, including level Ia, ipsilateral floor of mouth, sublingual gland, and mandibular lingual-side periosteum. Local mandibular resection was performed in two patients with gingival carcinoma. Following the confirmation of no residual tumor and no cervical lymph node metastases by intraoperative frozen-section pathology, submandibular gland flap was performed to reconstruct the oral defect, and sternocleidomastoid myocutaneous flap was used to reconstruct the floor of the mouth.

### Design and preparation of combined submandibular gland and sternocleidomastoid myocutaneous flap and reconstruction

During routine neck dissection, a horizontal skin incision was first made 2 cm below the mandible and carried down through the subcutaneous tissue and platysma. The submandibular gland was then dissected free of the surrounding structures cranially and caudally, up to the horizontal branch of the mandible and down to the level of the hyoid bone. During the preparation of the submandibular gland flap, the capsule of the submandibular gland was well-protected.

During level I neck dissection, all lymphatic tissue and fatty tissue at levels I to V were dissected out, and frozen-section and paraffin-section pathological examinations for cancer metastases were subsequently conducted.

The inferior border of the submandibular gland was released and separated from the digastric muscle. The proximal ends of the external maxillary artery and anterior facial vein were identified in the superior border of the deep surface of the posterior belly of the digastric muscle and were well-protected. The posterior border of the submandibular gland was then separated, and the distal ends of the external maxillary artery and anterior facial vein were both ligated with no damage to the marginal mandibular branches of the facial nerve. The superior border of the submandibular gland was released with its duct severed.

**Figure 1:**
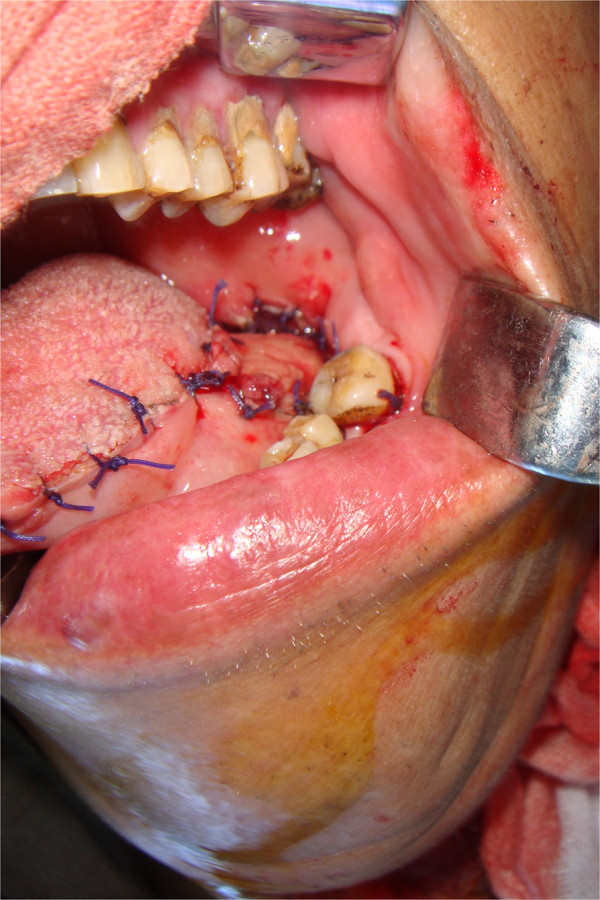
**Submandibular gland flap for reconstruction of root-of-tongue carcinoma, intraoperative view.**

Removal of the primary tumor was routinely performed. The released submandibular gland was then transferred to the site of the defect for reconstruction, and the proximal ends of the external maxillary artery and anterior facial vein were used as pedicles (Figure 1).

**Figure 2:**
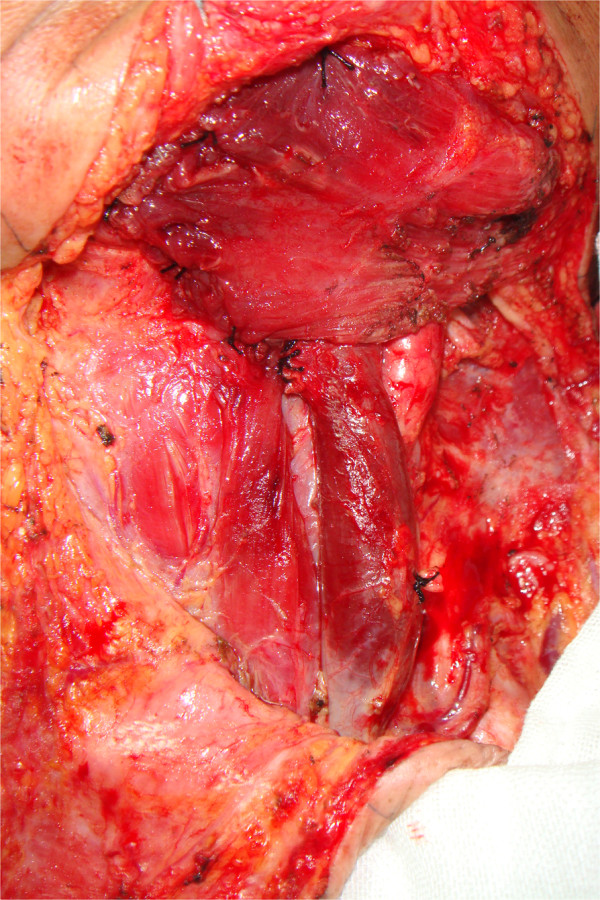
**Sternocleidomastoid myocutaneous flap for reconstruction of roof of mouth, intraoperative view.**

The clavicular end of the sternocleidomastoid was dissected to produce a sternocleidomastoid myocutaneous flap, and the clavicular end of sternocleidomastoid was then sutured to the anterior belly of the digastric muscle and mylohyoid muscle to reconstruct the floor of the mouth (Figure 2).

### Radiotherapy

Postoperative radiotherapy was conducted in two patients with tumors 4 × 3 cm using a linear accelerator (Siemens AG, Munich, Germany) with 6-MV x-rays. Patients were treated in a thermoplastic mask with isocenter irradiation. The non-irradiated area was shielded by an individualized low-melting-point lead shielding. Conventional fractionated radiotherapy was carried out at a dose of 60 Gy for the primary tumor site and a prophylactic dose of 44 Gy for cervical lymph nodes in 2-Gy fractions once daily five times per week.

### Clinical observations

Detailed clinical observations included: postoperative wound healing, complications, pathological examinations of submandibular, submental and cervical lymph nodes, postoperative contours of tongue, root of tongue and floor of mouth, postoperative contour and occlusion of mandible, restoration of breathing, phonation, swallowing, dehiscence and occlusion, and postoperative shoulder function, as well as relapse of primary lesion and cervical lymph nodes.

## Results

### Postoperative conditions

No patients died from the surgery. All submandibular gland flaps survived and no complications were observed. Postoperatively, the submandibular glands developed well-mucosalized surfaces and no significant pain from gland distension was noted. The operative incisions healed by first intention in all cases. Both intraoperative frozen sections and postoperative paraffin sections for all patients confirmed pathologically no cancer metastasis to any of the 27 dissected level I lymph nodes. Pathological examination also confirmed no lymphatic metastasis in the remaining levels in all cases.

### Appearance and function

**Figure 3:**
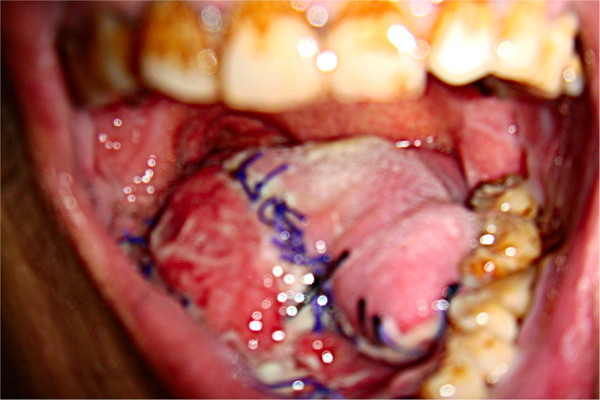
**Submandibular gland flap for reconstruction of tongue cancer.** One week postoperatively, surface mucosalization of the submandibular gland is bright red.

**Figure 4:**
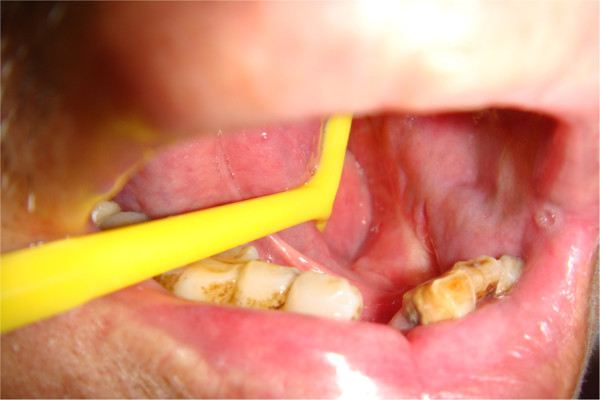
**Submandibular gland flap for reconstruction of gingival cancer.** Three months postoperatively, submandibular gland is essentially the same color as the oral mucosa.

After reconstruction, tongue function recovered well and the contour was pleasing. No significant impact on phonation was observed. For reconstruction of the tongue root, no negative impact on breathing or swallowing and no significant swelling were observed. Tongue movement was normal, with clear phonation. After floor-of-mouth reconstruction, no swelling or significant impact on swallowing, tongue movement, or phonation was observed. Mandibular reconstruction resulted in basically satisfactory appearance. Range of mouth-opening was normal, but slight malocclusion was observed. Swallowing and phonation were both normal. No postoperative shoulder dysfunction was found, indicating that separation of the sternocleidomastoid from the clavicle had not noticeably altered shoulder function (Figures 3 and 4).

### Follow-up

Postoperative follow-up began at the completion of treatment and ended 31 May 2013. No patient was lost to follow-up. During the follow-up period of 12 to 28 months, none of the patients developed local recurrence or lymphatic or distant metastases.

## Discussion

To date there has been no consensus about preservation of the submandibular gland during neck dissection in oral cavity and oropharyngeal cancers. Traditional surgical treatment includes excision of the submandibular gland and the surrounding lymphatic tissue in addition to removal of the primary lesion. Submandibular glands are frequently excised as part of neck dissection. With the development of functional surgery, an increasing number of investigations have demonstrated that cancer metastasis to the submandibular gland is quite rare and that it is feasible and oncologically safe to preserve the submandibular gland with no involvement of cancer metastases during neck dissection followed by close postoperative follow-up. Whether to preserve the submandibular gland depends on the following conditions: (1) whether there are intraglandular submandibular lymph nodes; (2) whether it is involved in the primary cancer; and (3) whether the vessels of the submandibular gland are left intact during level I neck dissection.

The existence of intraglandular lymph nodes within the submandibular gland currently remains controversial [5, 6]. Because of their doubts about the existence of intraglandular lymph nodes, Rouviere and Tobies as well as DiNardo performed pathological studies to investigate intraglandular lymph nodes using human submandibular gland tissue sections from cadavers. In both studies, no intraglandular lymph nodes were identified in the submandibular gland samples [4, 6]. Other investigations have reported similar results [13, 14]. Even if there were intraglandular lymph nodes, the lymphatic drainage of intraglandular lymph nodes might not be truly identical to that of the lymph nodes outside the submandibular gland capsule because of the difference in anatomic structure between the submandibular gland and submandibular triangle. Moreover, whether lymph from surrounding lymphatic tissue can be directed into intraglandular lymph nodes is less clear and requires further study. Conversely, numerous studies have suggested that although metastatic lymph nodes rarely invade the gland, metastasis to level Ib occurs frequently in oral cavity cancer [15, 16]. Hence, although there is no universal consensus about the existence of intraglandular lymph nodes thus far, it is generally believed that they do not exist within the submandibular gland.

Head and neck carcinoma, especially oral cancer, seldom metastasizes to the salivary glands. If it occurs, the parotid gland is more likely to be affected than the submandibular gland [17]. Although oral cancer most often metastasizes to neck lymph nodes and levels I, II, and III are the most frequently involved [4], the involvement of the submandibular gland in oral cancers is extremely rare [5, 6]. In a study investigating whether and how head and neck squamous carcinoma metastasizes to the submandibular gland, Spiegel *et al*. demonstrated that, because of the absence of intraglandular lymph nodes among 196 examined submandibular glands, in no case did a squamous cell carcinoma metastasize to the submandibular gland [8]. The authors found that the submandibular gland was involved only in cases in which the primary tumor was in close proximity to the gland, or when metastasis to level I of the neck had occurred by extension from a locally affected lymph node into the submandibular gland. Byeon *et al*. reported in 2009 that of 201 cases of oral cavity squamous cell carcinoma, only two (1%) had carcinomatous involvement in the submandibular gland through direct extension from a primary lesion and no submandibular glands showed pathological evidence of isolated metastasis or local extension of metastatic lymph nodes. Based on their study findings, the authors thought that it might be feasible and safe to preserve an oncologically sound submandibular gland during neck dissection in patients with early-stage oral cancer [18]. Other studies revealed similar results, demonstrating that submandibular gland metastasis from head and neck primary squamous cell carcinoma was extremely rare and that direct invasion as a primary mechanism of metastasis led to most of the metastases [6, 7, 9, 10]. These studies concluded that preservation of the submandibular gland might be oncologically safe and feasible unless there was evidence of direct invasion of the gland or close proximity of the cancer to it. Taken together, head and neck carcinoma, especially early-stage oral cancer, can be characterized by extremely rare metastasis to the submandibular glands. This metastasis profile provides a solid oncological basis for the use of the preserved submandibular gland as reconstruction material.

Submandibular glands are located in level Ib, surrounded by rich lymphatic tissue. The important question is whether this preparatory procedure in creation of a submandibular gland flap is technically feasible; that is whether all lymph nodes in level Ib can be eradicated while leaving the submandibular gland intact for reconstruction during neck dissection. More recently, Dhiwakar *et al*. performed an anatomic pathological study to determine whether all lymph nodes in level Ib can be extirpated without removing the submandibular gland [19]. The authors reported that complete removal of lymph nodes in level Ib was achieved in all 30 neck dissections and that the submandibular gland and surgical bed contained no residual lymph nodes. They concluded that, in suitable cases, it was technically feasible to remove all level Ib lymph nodes and preserve the submandibular gland.

The next question is whether the submandibular gland can survive and maintain its integrity and function following transplantation. Survival of the transplanted submandibular gland is crucial to this reconstruction method. It is clearly unwise to select a submandibular gland as a candidate for reconstruction material if it fails to survive following transplantation. To prevent xerostomia and protect the submandibular gland from radiation damage, Seikaly *et al*. performed surgical transfer of the submandibular gland to the submental space outside the proposed radiation field in patients with head and neck carcinoma and carried out long-term postoperative follow-up. The authors reported that the transferred submandibular glands survived and maintained salivary function for statistically significant prevention of xerostomia, and that there were no disease recurrences on the side of the transferred gland or in the submental space [11]. Al-Qahtani K *et al*. conducted a prospective clinical trial in which the submandibular gland was similarly transferred to the submental space. Their results also suggested that the submandibular gland can survive with its function preserved following surgical transplantation [20].

Therefore, based on these above-mentioned findings, we believe that using the submandibular gland flap to reconstruct oral defects is oncologically safe and feasible in patients with early oral cavity and oropharyngeal cancers. In this study, we preserved the ipsilateral submandibular gland during neck dissection and reconstructed oral defects using the transferred submandibular gland in eight patients. Surgeries were successful in all eight patients and there were no resulting deaths. All submandibular gland flaps survived with well-mucosalized surfaces. No complications were observed. The operative incisions healed by primary intention in all eight cases. Twenty-seven lymph nodes were dissected out of level I in all patients. Pathological examinations of intraoperative frozen sections and postoperative paraffin sections confirmed no cancer metastases to any of the 27 lymph nodes. No cancer metastasis was found in the remaining levels in any patient. No patient developed local relapse or lymphatic or distant metastasis during the follow-up period of 12 to 28 months.

As a reconstruction material, the submandibular gland has several major advantages. First, its abundant blood supply and a consistent, bulky vascular pedicle make it difficult for the flap to develop necrosis following transplantation. Second, a submandibular gland flap is easy to obtain and prepare. Its preparation requires no special surgical skills and can be completed during neck dissection. A submandibular gland flap can be easily prepared for use upon completion of neck dissection. The key points of the operation are to use care to keep the capsule of the submandibular gland intact and to protect the proximal and distal ends of the external maxillary artery and anterior facial vein during preparation. Third, this method does not require vascular anastomosis. The relatively simple anatomic structure of the submandibular gland facilitates the preparation of a submandibular gland flap in less time than conventional methods. Additionally, neck dissection and flap preparation can be performed as a single-stage procedure, which simplifies the operation and reduces operative duration. Finally, the simplified procedure and shorter duration result in fewer traumas, lower complication rates, and, consequently, decreased surgical risk.

Given that a submandibular gland flap cannot completely fill the oral defect because of its limited size, in this study we conducted a combined reconstruction using a submandibular gland flap for oral and oropharyngeal defects and a sternocleidomastoid myocutaneous flap for floor-of-mouth defects. Similar to a submandibular gland flap, a sternocleidomastoid myocutaneous flap is easy to obtain and prepare and does not require a complex surgical procedure. Therefore, this combined reconstructive technique can greatly minimize surgical trauma and operating time.

These advantages suggest that this reconstructive technique may be the preferred treatment option in suitable cases, especially in older aged patients with complicated systemic disease. In the present study, all eight patients had comorbid cardiovascular disease, cerebrovascular disease, diabetes, or chronic respiratory disease that made our patients poor candidates for lengthy and risky surgical procedures. Thus, we performed reconstructive surgery using combined submandibular gland and sternocleidomastoid myocutaneous flaps.

Our preliminary results were encouraging. Surgery was successful in all eight patients, with no surgical deaths and no complications. Compared with the conventional method we previously used, this combined reconstruction method shortened operative duration by about 30% on average. The cosmetic and functional outcomes were satisfactory in all eight patients. Reconstruction of the tongue resulted in good recovery of tongue function, a pleasing contour, and no significant impact on phonation. After reconstruction of the tongue root, no negative impact on breathing or swallowing and no significant swelling were observed. Tongue movement was normal and phonation was clear. Floor-of-mouth reconstruction resulted in no swelling and no significant impact on swallowing, tongue movement, or phonation. Appearance after mandibular reconstruction was basically satisfactory; range of mouth opening was normal although there was slight malocclusion. However, swallowing and phonation were both normal. No significant postoperative shoulder dysfunction was noted, suggesting that the separation of the sternocleidomastoid from the clavicle did not greatly alter this. Appropriate restoration of oral function and facial aesthetics greatly improved the quality of life of these older aged patients.

Despite its advantages, there are several disadvantages to this reconstruction method. First, safety is an underlying concern. To the best of our knowledge, no large multicenter clinical study - particularly a prospective study - has been conducted to prove that intraglandular lymph nodes do not exist within the submandibular glands or that the submandibular gland cannot be involved in oral cavity and oropharyngeal cancers through lymphatic metastasis. Thus, only in cases in which no neck or level I lymphatic metastases are confirmed pathologically should the submandibular gland be used for reconstruction. This limits the clinical application of this technique in a considerable number of patients. A second disadvantage is the limited length of the vascular pedicle. If the proximal ends of the anterior facial artery and vein are used as vascular pedicles, the submandibular gland flap can extend only 2.0 ± 7.0 cm away from donor site because of the length limitation of the pedicle [21]. If the distal ends of the anterior facial artery and vein are used as vascular pedicles, the reach of this flap can be improved to some extent with blood supply to the transferred submandibular gland from the reverse flow of the external maxillary artery and anterior facial vein. A third disadvantage is the reconstruction range. The maximal reconstruction range is equal to the maximal cross-section of the submandibular gland [22], so the submandibular gland flap cannot cover a larger defect [15]. A final disadvantage concerns submandibular gland secretions. It has been reported that the denervated submandibular gland maintains long-term secretory function [13, 16]. In this reconstruction method, the transferred submandibular gland is denervated once it is completely detached from surrounding tissue during flap preparation, and about half of the submandibular gland is exposed in the oral cavity after completion of the reconstruction. Little is known about whether the exposure in the oral cavity has an impact on secretory function of the denervated gland. If the exposure has little or no impact and salivary function after denervation is preserved, the submandibular gland may develop swelling and pain after reconstructive surgery since the submandibular ducts have been severed during flap preparation. In this study, because of the limitations in lengths of vascular pedicles and ranges of reconstruction, we performed the reconstruction only for oral cancers in the posterior two-thirds of the tongue and both sides of the floor of mouth, gingival cancer adjacent to the molars, and oropharyngeal cancer close to the tongue. The maximal reconstruction range was 6 × 5 cm.

We note several limitations to this study. The number of patients in this study was relatively small and the follow-up period relatively short, and thus far no prospective randomized comparative clinical study has been conducted to confirm the feasibility of this combined reconstruction technique in a large number of patients. Therefore, the long-term clinical effects, safety, and application scope of this technique need to be further evaluated with adequate statistical power in future clinical practice.

## Conclusions

Skin flap, musculocutaneous flap, and osteo-myocutaneous flap are conventional methods of postoperative reconstruction in patients with oral cavity and oropharyngeal cancers. However, older aged patients with oral cavity and oropharyngeal cancers tend to have comorbid conditions. Older aged patients with this complex medical condition have poor surgical tolerance and thus cannot undergo a lengthy and potentially risky surgical operation. Our combined reconstruction technique with submandibular gland flap and sternocleidomastoid myocutaneous flap has the advantages of being easier to perform, causing less surgical injury and only minor impact on contour, and greatly reducing operation time and surgical risk. This technique can facilitate the restoration of oral function and facial aesthetics and greatly improve the quality of life of older aged patients. Hence, this technique can provide a new and safe reconstruction option for older aged cancer patients with complex medical conditions. If we can verify at the molecular level the absence of intraglandular lymph nodes and that there is no lymphatic metastasis to the submandibular glands, this combined reconstruction technique may gain popularity.

## References

[1] Kubo T, Osaki Y, Hattori R, Kanazawa S, Hosokawa K (2013). Reconstruction of through-and-through oromandibular defects by the double-skin paddle fibula osteocutaneous flap: can the skin paddle always be divided?. J Plast Surg Hand Surg.

[2] Rudes M, Bilić M, Jurlina M, Prgomet D (2012). Pectoralis major myocutaneous flap in the reconstructive surgery of the head and neck - our experience. Coll Antropol.

[3] Stott-Miller M, Chen C, Chuang SC, Lee YC, Boccia S, Brenner H, Cadoni G, Dal Maso L, La Vecchia C, Lazarus P, Levi F, Matsuo K, Morgenstern H, Müller H, Muscat J, Olshan AF, Purdue MP, Serraino D, Vaughan TL, Zhang ZF, Boffetta P, Hashibe M, Schwartz SM (2012). History of diabetes and risk of head and neck cancer: a pooled analysis from the international head and neck cancer epidemiology consortium. Cancer Epidemiol Biomarkers Prev.

[4] Rouviere H, Tobies MJ (1938). Trans: Anatomy of the Human Lymphatic System.

[5] Haagensen CD (1972). The Lymphatics in Cancer.

[6] DiNardo LJ (1998). Lymphatics of the submandibular space: an anatomic, clinical, and pathologic study with applications to floor-of-mouth carcinoma. Laryngoscope.

[7] Ebrahim AK, Loock JW, Afrogheh A, Hille J (2011). Is it oncologically safe to leave the ipsilateral submandibular gland during neck dissection for head and neck squamous cell carcinoma?. J Laryngol Otol.

[8] Spiegel JH, Brys AK, Bhakti A, Singer ML (2004). Metastasis to the submandibular gland in head and neck carcinomas. Head Neck.

[9] Razfar A, Walvekar RR, Melkane A, Johnson JT, Myers EN (2009). Incidence and patterns of regional metastasis in early oral squamous cell cancers: feasibility of submandibular gland preservation. Head Neck.

[10] Chen TC, Lo WC, Ko JY, Lou PJ, Yang TL, Wang CP (2009). Rare involvement of submandibular gland by oral squamous cell carcinoma. Head Neck.

[11] Seikaly H, Jha N, Harris JR, Barnaby P, Liu R, Williams D, McGaw T, Rieger J, Wolfaardt J, Hanson J (2004). Long-term outcomes of submandibular gland transfer for prevention of postradiation xerostomia. Arch Otolaryngol Head Neck Surg.

[12] Geerling G, Honnicke K, Schröder C, Framme C, Sieg P, Lauer I, Pagel H, Kirschstein M, Seyfarth M, Marx AM, Laqua H (1999). Quality of salivary tears following autologous submandibular gland transplantation for severe dry eye. Graefes Arch Chin Exp Ophthalmol.

[13] Sinha UK, Ng M (1999). Surgery of the salivary glands. Otolaryngol Clin North Am.

[14] Rosti G, Callea A, Merendi R, Beccati D, Tienghi A, Turci D, Marangolo M (1987). Metastases to submaxillary gland from breast cancer: case report. Tumori.

[15] Junquera L, Albertos JM, Ascani G, Baladrón J, Vicente JC (2000). Involvement of the submandibular region in epidermoid carcinoma of the mouth floor. Prospective study of 31 cases. Minerva Stomatol.

[16] Basaran B, Ulusan M, Orhan KS, Gunes S, Suoglu Y (2013). Is it necessary to remove submandibular glands in squamous cell carcinomas of the oral cavity?. Acta Otorhinolaryngol Ital.

[17] Vessecchia G, Di Palma S, Giardini R (1995). Submandibular gland metastasis of breast carcinoma: a case report and review of the literature. Virchows Arch.

[18] Byeon HK, Lim YC, Koo BS, Choi EC (2009). Metastasis to the submandibular gland in oral cavity squamous cell carcinomas: pathologic analysis. Acta Otolaryngol.

[19] Dhiwakar M, Ronen O, Malone J, Rao K, Bell S, Phillips R, Shevlin B, Robbins KT (2011). Feasibility of submandibular gland preservation in neck dissection: a prospective anatomic-pathologic study. Head Neck.

[20] Al-Qahtani K, Hier MP, Sultanum K, Black MJ (2006). The role of submandibular salivary gland transfer in preventing xerostomia in the chemoradiotherapy patient. Oral Surg Oral Med Oral Pathol Oral Radiol Endod.

[21] Martin D, Pascal JF, Baudet J, Mondie JM, Farhat JB, Athoum A, Le Gaillard P, Peri G (1993). The submental island flap: a new donor site anatomy and clinical applications as a free or pedicled flap. Plast Reconstr Surg.

[22] Sterne GD, Januszkiewicz JS, Hall PN, Bardsley AF (1996). The submental island flap. Br J Plast Surg.

